# The efficacy of platelet-rich plasma applicated in spinal fusion surgery: A meta-analysis

**DOI:** 10.3389/fsurg.2022.924753

**Published:** 2022-09-23

**Authors:** Hongwei Yu, Zhaohong Zhou, Bin Yu, Tianwei Sun, Qiong Tang, Yutao Jia

**Affiliations:** ^1^ School of Medicine, Nankai University, Tianjin, China; ^2^ Department of Spinal Surgery, Tian-jin Union Medical Centre, Nankai University People’s Hospital, Tianjin, China; ^3^ Department of Respiratory Medicine, Tian-jin Union Medical Centre, Nankai University People’s Hospital, Tianjin, China

**Keywords:** fusion rate, platelet-rich plasma (PRP), autologous growth factors, spinal surgery, spinal fusion

## Abstract

**Objective:**

The purpose of this meta-analysis is to evaluate the effect of the application of platelet-rich plasma (PRP) in spinal fusion surgery on the fusion rate of the spine.

**Methods:**

A comprehensive search of the PubMed, Embase, Cochrane Library, and Science Direct databases was conducted to identify randomized control trials (RCTs) or observational cohort studies that evaluated the efficacy and safety of PRP in spinal fusion. Data on final fusion rate, changes in the visual analog scale (VAS), estimated blood loss (EBL), and operative time was collected from the eligible studies for meta-analysis. Patients were divided into PRP and non-PRP groups according to whether PRP was used during the spinal fusion procedure.

**Results:**

According to the selection criteria, 4 randomized controlled trials and 8 cohort studies with 833 patients and 918 levels were included. The outcomes indicated that PRP application is associated with a lower fusion rat (OR = 0.62, 95% CI: (0.43, 0.89), *P* = 0.009) at final follow-up (>24 months). Subgroup analysis showed a lower rate of spinal fusion in the PRP group compared to the non-PRP group (OR = 0.35, 95% CI: (0.21, 0.58), *P* < 0.001) when spinal fusion was assessed using only anterior-posterior radiographs. When the bone graft material was a combination of autologous bone + artificial bone, the spinal fusion rate was lower in the PRP group than in the non-PRP group (OR = 0.34, 95% CI: (0.16, 0.71), *P* = 0.004). The PRP and non-PRP groups showed no significant differences in VAS changes at the 24th postoperative month (WMD = 0.36, 95% CI: (−0.37, 1.09), *P* = 0.33); Application of PRP does not reduce the estimated blood loss (WMD = −86.03, 95% CI: (−188.23, 16.17), *P* = 0.10). In terms of operation time, using PRP does not prolong operation time (WMD = −3.74, 95% CI: (−20.53, 13.04), *P* = 0.66).

**Conclusion:**

Compared with bone graft fusion alone, PRP cannot increase the rate of spinal fusion. Inappropriate methods of spinal fusion assessment or mixing PRP with artificial/allograft bone may have been responsible for the lower rate of spinal fusion in the PRP group.

**Systematic Review Registration:**

doi: 10.37766/inplasy2022.5.0055

## Introduction

Spinal fusion is an important method used to treat degenerative and traumatic diseases of the spine. Spinal non-fusion refers to the failure of bridging of adjacent vertebrae more than 1 year after surgery (1). Failure of spinal fusion will result in pseudoarthrosis, a common complication after spinal surgery. The formation of a pseudarthrosis often leads to loss of correction, recurrence of deformity, instability of the lumbar spine, low back pain with activity or weight bearing, or neurological symptoms (2). The prevalence of pseudarthrosis reported in the literature ranges from 0% to 56% (3). However, since many patients with pseudarthrosis remain asymptomatic, the true incidence may be underestimated by the literature. The use of bone graft extenders, such as bone morphogenetic proteins (BMPs) or platelet-rich plasma (PRP), has been considered to address this problem (4). Several studies (5–7) have shown that BMPs can improve spinal fusion rates. However, the possible side effects of BMPs, including inflammation, heterotopic bone formation, neck swelling, and radiculitis, have been reported (8, 9). In 2008, the FDA Public Health Notification published an alert regarding safety concerns for BMPs, which led to a gradual decline in their use (10). Therefore, an effective and safe method is needed to increase the rate of bone fusion after spinal fusion surgery.

Platelet activation can produce a variety of autologous growth factors (AFGs), such as platelet-derived growth factor (PDGF) and transforming growth factor-beta (TGF-b) (11). These growth factors promote mitogenesis in fibroblasts, osteoblasts, and mesenchymal cells by stimulating DNA synthesis, allowing them to proliferate and secrete more growth factors, and these cells then differentiate into osteoblasts. In addition, these growth factors also have a chemotactic effect on undifferentiated stem cells (12, 13). Therefore, high concentration of PRP in the fusion levels shows potential as an excellent osteoinductive agent or mitotic factor, which helps to promote bone fusion (14). At present, the technology of producing ultraconcentrated platelets has been produced and promoted as a result of its generated osteogenic action. However whether PRP promotes spinal fusion is inconclusive, even though many meta-analyses currently exist to resolve this controversy. For example, the pooled results of Saran et al. showed no difference in the final spinal fusion rate with the combination of PRP and autologous bone compared to autologous bone graft alone (15). The pooled results of Yolcu et al. (16) showed that the addition of PRP to the spinal fusion process decreased the final spinal fusion rate. Therefore, the objective of this meta-analysis is to re-evaluate the efficacy of PRP, which can aid in the decision-making process regarding the use of PRP in spinal fusion surgery.

## Methods

The guidelines used for this systematic review and meta-analysis were the Preferred Reporting Items for Systematic Reviews and Meta-Analyses (PRISMA) (17). The protocol for this review was registered on the International Platform of Registered Systematic Review and Meta-analysis Protocols database with the registration number INPLASY202250055 and DOI number 10.37766/inplasy2022.5.0055.

### Search strategy and study selection

A comprehensive literature search was performed through the following databases: PubMed, EMBASE, Cochrane Library, and ScienceDirect. We identified relevant articles published up to 1 May 2022 without language limitations. Studies were found using the following keywords: platelet-rich plasma, PRP, platelet gel, spinal fusion, bone inducer, and bone extenders. Two independent investigators screened eligible studies and reviewed references of the included studies to identify additional articles. When consensus could not be reached, a third reviewer was consulted.

### Select strategy

The inclusion and exclusion criteria of studies followed PICOS principles. (1) Participants: Patients with spinal degenerative or traumatic diseases requiring spinal fusion treatment. (2) Interventions: Patients in whom the bone graft material used for spinal fusion is a mixture of grafted bone and PRP. (3) Comparisons: Patients whose bone graft material used for spinal fusion is bone graft alone. (4) Outcomes: Studies should include at least one of the following data: final spinal fusion rate, final changes of VAS, EBL, and operative time. (5) Study design: Observational studies and randomized control trials were eligible. Case reports, case series, commentaries, practice guidelines, systematic review,s an metaanalysiss were excluded.

### Data extraction

The following data were extracted from the included studies: (1) study design: first author, country, publication time, and study type; (2) sample demographics: number of patients and fused levels, follow-up time, age, and sex; (3) fusion details: surgical procedure, bone graft material, imaging modalities for fusion assessment; (4) PRP preparation, formulation, and application methods; and (5) analysis variables: final spinal fusion rate (at least 24 months), final changes of VAS, EBL (excluding the amount of blood consumed for PRP preparation), and operative time. Successful spinal fusion is defined as the presence of bridging bone remodeling between the vertebral bodies or between the bilateral posterolateral intertransverse on static radiographs (anterior-posterior radiographs or CT) (18), or adjacent vertebrae translation <3 mm, angle <5° onflexion-extensionn radiographs (18).

### Assessment of risk of bias

Two researchers independently assessed the risk of bias in randomized trials using the revised Cochrane Risk of Bias tool (RoB-2) (19) and the risk of bias in non-randomized trials using Risk of Bias In Non-Randomized Studies of Interventions (ROBINS-I) (20). Sensitivity analysis was performed by excluding a single study from each study and reanalyzing the data. Publication bias was detected by the Funnel diagram. Sensitivity analysis and publication bias analysis were implemented using RevMan 5.3.

### Statistical analysis

The continuous data were calculated by weighted mean differences (WMDs) with 95% confidence intervals (CIs), and dichotomous variables were calculated by using odds ratios (ORs) with 95% confidence intervals (CIs). Statistical heterogeneity was calculated by using a chi-square test and *I*^2^ test. When *I*^2^ ≤ 50%, we performed a fixed-effect model for the meta-analysis. Otherwise, the random-effect model was performed. To investigate the impact of fusion assessment tools and the use of different bone grafting materials on spinal fusion rate, we also performed subgroup analysis. The meta-analysis was performed using RevMan 5.3 for Windows (Cochrane Collaboration, Oxford, UK). If the result of the meta-analysis was a probability of *P* < 0.05, it was statistically significant.

## Results

### Search results

**Figure 1:**
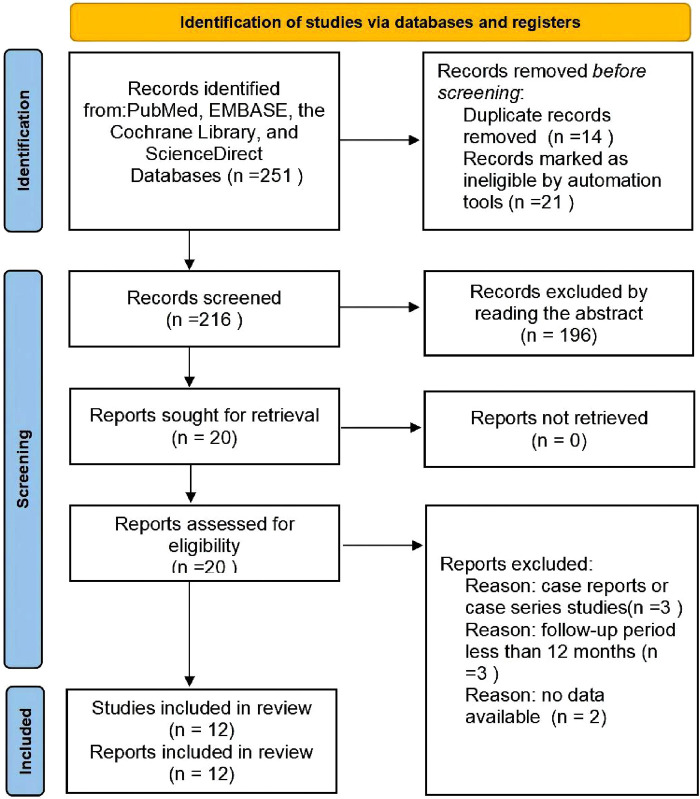
PRISMA flowchart.

A total of 251 articles from PubMed, EMBASE, the Cochrane Library, and ScienceDirect were initially identified. PubMed (*n* = 132), EMBASE (*n* = 62), ScienceDirect (*n* = 53), and the Cochrane Library (*n* = 4). 231 studies were directly excluded by screening the titles and abstracts. 20 studies underwent a comprehensive full-text analysis. Finally, 12 studies met the inclusion criteria and were included in this meta-analysis. The flow diagram of the search strategy is summarized in Figure 1.

### Study characteristics and risk of bias

The eligible studies included 4 randomized control trials and 8 cohort studies. A total of 833 patients and 918 levels were involved in the 12 eligible studies. The PRP group included 364 patients and 401 levels, and the non-PRP group included 469 patients and 517 levels. The characteristics of the included studies are presented in Table 1. The imaging modalities and successful fusion or pseudarthrosis criteria for each study are shown in Table 2. The preparation methods, formulations, and usage of PRP are summarized in Table 3. The results of the quality evaluation of randomized controlled studies and non-randomized controlled studies are summarized in Tables 4, 5.

**Table 1:** Demographic characteristics of the included studies.

PRP/non-RP									
Study ID	Study type	Country	Number of participants	Fused levels	Age (Mean), year	Gender (F)	Surgical approach	Type of bone graft	Follow-up (months)
Tsai 2009	RCT	Taiwan	34/33	34/33	59.8/63.3	6:27	PLF	Autologous bone + artificial bone	28.5/27.6
Acebal 2011	Prospective non-randomized	Spain	67/40	67/40	57/59	24:16	PLF	Autologous bone + artificial bone	24
Weiner 2003	Retrospective cohort	United States	32/27	32/27	61/56	11:16	PLF	Autologous bone	24
Carreon 2005	Retrospective cohort	United States	76/76	76/76	50.6/49.9	40:36	PLF	Autologous bone	32/37
Castro 2004	Prospective cohort	United States	22/62	28/76	47/49	21:41	TLIF	Autologous bone	34/41
Hee 2003	Prospective cohort	Singapore	23/111	23/111	44.3/47.7	42:69	TLIF	Autologous bone	24
Kubota 2018	Retrospective cohort	Japan	11/9	11/9	59.4/63.3	4:5	TLIF	Autologous bone	24
Kubota 2019	RCT	Japan	25/25	32/35	65.1/65.3	14:11	PLF	Autologous bone	24
Jenis 2006	Prospective non-randomized	United States	15/22	22/32	40.3/41.4	14:8	ALIF	Autologous bone	25.7/24.3
Sys 2011	RCT	Belgium	19/19	19/19	74.9/76	12:7	PLIF	Autologous bone	24
Feiz-erfan 2007	RCT	United States	25/25	42/39	/	/	ACDF	Allogeneic bone	24
Hartmann 2009	Retrospective	Germany	15/20	15/20	43.7/39.8	13:7	ALIF	Autologous bone	12
	Cohort								

PLF, posterolateral fusion; PLIF, posterior lumbar interbody fusion; ALIF, anterior lumbar interbody fusion; TLIF, transforaminal lumbar interbody fusion; ACDF, anterior cervical decompression and fusion.

**Table 2:** Criteria and evaluation methods for successful spinal fusion in each study.

Study ID	Imaging modalities	Criteria for successful fusion
Tsai 2009	CT/FE	FE: <5 degree of angular motion or <2 mm of translation at the operated level;
		CT: bridging bone remodeling occurringbetween the bilateral posterolateral intertransverse
Acebal 2011	AP	Bridging bone remodeling occurring between the bilateral posterolateral intertransverse
Weiner 2003	AP	Bridging bone remodeling occurring between the bilateral posterolateral intertransverse
Carreon 2005	CT	Bridging bone remodeling occurring between the bilateral posterolateral intertransverse
Castro 2004	AP	Bridging bone remodeling occurring between the vertebral body or bridging bone remodeling occurring between the bilateral posterolateral intertransverse
Hee 2003	AP	Bridging bone remodeling occurring between the vertebral body or bridging bone remodeling occurring between the bilateral posterolateral intertransverse
Kubota 2018	CT	Bridging bone remodeling occurring between the vertebral body
Kubota 2019	CT	Bridging bone remodeling occurring between the unilateral posterolateral intertransverse
Jenis 2006	CT	Bridging bone remodeling occurring between the vertebral body
Sys 2011	CT	Bridging bone remodeling occurring between the vertebral body
Feiz-erfan 2007	PE	No significant angular motion (no more than 2°)
Hartmann 2009	CT	Bridging bone remodeling occurring between the vertebral body

**Table 3:** PRP preparation techniques, formulations and usage.

Study ID	Source	Preparation technology	Preparation method	Core product	Platelet concentration	Activators	Start preparation time	Drug delivery methods	Implantation site
Tsai 2009	Peripheral vein blood/NA	Gravity centrifugation	NA	NA	NA	CaCl + Thrombin	Intraoperative	Mixed with bone graft	Between the bilateral posterolateral intertransverse
Acebal 2011	Peripheral vein blood/100 ml	Gravity centrifugation	Two-step method	brownish-yellow layer	NA	Thrombin	NA	Mixed with bone graft	Between the bilateral posterolateral intertransverse
Weiner 2003	Peripheral vein blood/450 ml	Gravity centrifugation	Two-step method	brownish-yellow layer	6-fold	Thrombin	Intraoperative	Mixed with bone graft	Between the bilateral posterolateral intertransverse
Carreon 2005	Peripheral vein blood/500 ml	Gravity centrifugation	Two-step method	brownish-yellow layer	NA	Thrombin	Intraoperative	Mixed with bone graft	Between the bilateral posterolateral intertransverse
Castro 2004	Peripheral vein blood/450 ml	Gravity centrifugation	Two-step method	brownish-yellow layer	3.5 fold	Thrombin	Intraoperative	Mixed with bone graft	Between the vertebral bodies
Hee 2003	Peripheral vein blood/450 ml	Gravity centrifugation	Two-step method	brownish-yellow layer	4.89 fold	CaCl + Thrombin	Intraoperative	Mixed with bone graft	Between the vertebral bodies
Kubota 2018	Peripheral vein blood/450 ml	Gravity centrifugation	Two-step method	brownish-yellow layer	3–6 fold	CaCl + Thrombin	Intraoperative	Mixed with bone graft	Between the vertebral bodies
Kubota 2019	Peripheral vein blood/450 ml	Gravity centrifugation	Two-step method	brownish-yellow layer	NA	CaCl + Thrombin	Intraoperative	Mixed with bone graft	Between the bilateral posterolateral intertransverse
Jenis 2006	Peripheral vein blood/450 ml	Gravity centrifugation	Two-step method	brownish-yellow layer	NA	CaCl + Thrombin	Intraoperative	Mixed with bone graft	Between the vertebral bodies
Sys 2011	Peripheral vein blood/NA	Gravity centrifugation	NA	NA	3.3-fold	CaCl + Thrombin	Intraoperative	Mixed with bone graft	Between the vertebral bodies
Feiz-erfan 2007	Peripheral vein blood/NA	Gravity centrifugation	NA	NA	NA	CaCl + Thrombin	NA	Mixed with bone graft	Between the vertebral bodies
Hartmann 2009	Peripheral vein blood/450 ml	Gravity centrifugation	Two-step method	brownish-yellow layer	NA	Thrombin	Intraoperative	Mixed with bone graft	Between the vertebral bodies

**Table 4:** The risk of bias in non-randomized trials using risk of bias in Non-randomized studies of interventions (ROBINS-I).

Study ID	Bias due to confounding	Bias in selection of participants	Bias in classification of interventions	Bias due to deviations from intended interventions	Bias due to missing data	Bias in measurement of outcomes	Bias in selection of reported results	Overall risk of bias
Acebal 2011	Serious	Low	Low	Moderate	Low	Low	Moderate	Moderate
Weiner 2003	Serious	Moderate	Low	Low	Moderate	Low	Low	Moderate
Carreon 2005	Moderate	Low	Low	Serious	Low	Serious risk	Low	Serious
Castro 2004	Serious	Moderate	Low	Low	Low	Low	Low	Moderate
Hee 2003	Moderate	Low	Serious	Moderate	Low	Moderate	Low	Moderate
Kubota 2018	Moderate	Moderate	Low	Low	Moderate	Moderate	Low	Moderate
Jenis 2006	Serious	Low	Low	Serious	Low	Low	Moderate	Serious
Hartmann 2009	Moderate	Low	Moderate	Low	Serious	Low	Low	Moderate

**Table 5:** The risk of bias in randomized trials using the revised cochrane risk of bias tool (RoB-2).

Study ID	Bias arising from the randomization process	Bias due to deviations from intended intervention	Bias due to missing outcome data	Bias in measurement of the outcome	Bias in selection of the reported result	Overall risk of bias
Tsai 2009	Moderate	Low	Moderate	Low	Low	Moderate
Kubota 2019	Low	Low	Low	Low	Low	Low
Sys 2011	Moderate	Low	Low	Low	Low	Low
Feiz-erfan 2007	Low	Moderate	Low	Moderate	Low	Moderate

### Final fusion rate (at least 24 months)

**Figure 2:**
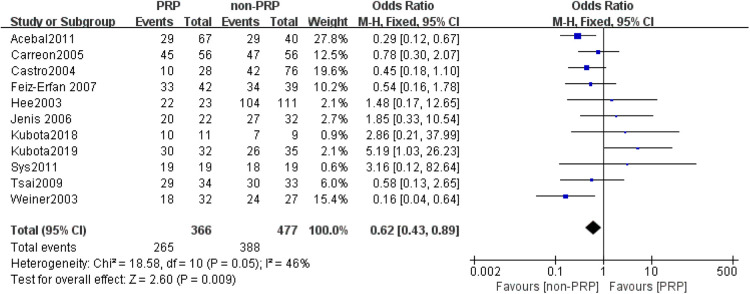
Forest plot of the final fusion rate (at least >2 years).

**Figure 3:**
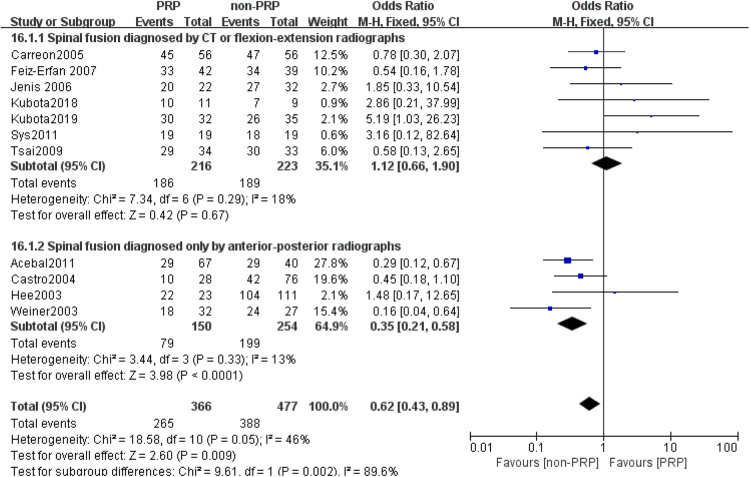
Subgroup analysis according to the spinal fusion evaluation methods.

**Figure 4:**
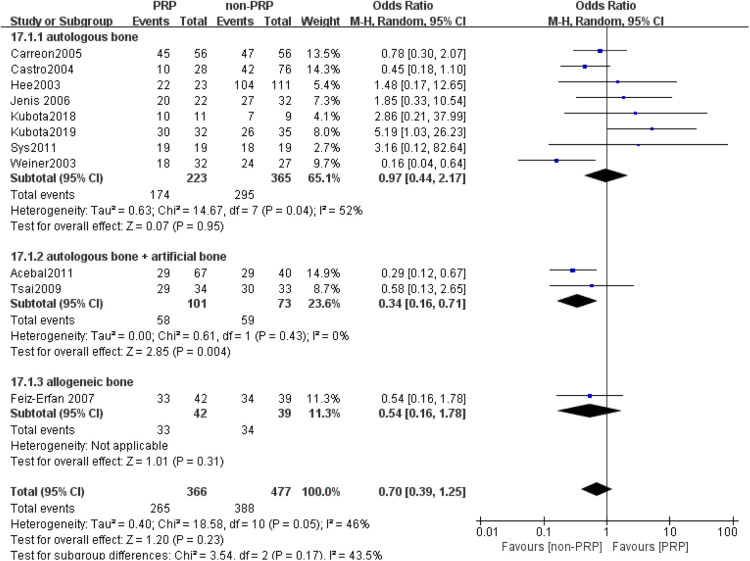
Subgroup analysis according to graft bone types.

A total of 11 studies (21–31) reported final fusion rate in 851 levels (369 levels in the PRP group and 482 levels in the non-PRP group). The outcomes indicated that PRP application is associated with a lower fusion rate, and the difference was statistically significant (OR = 0.62, 95% CI: (0.43, 0.89), *P* = 0.009), as shown in Figure 2. Subgroup analysis showed a lower rate of spinal fusion in the PRP group compared to the non-PRP group (OR = 0.35, 95% CI: (0.21, 0.58), *P* < 0.001) when spinal fusion was assessed using only anterior-posterior radiographs (Figure 3). And when the bone graft material was a combination of autologous bone + artificial bone (Figure 4), the spinal fusion rate was lower in the PRP group than in the non-PRP group (OR = 0.34, 95% CI: (0.16, 0.71), *P* = 0.004).

### VAS changes

**Figure 5:**
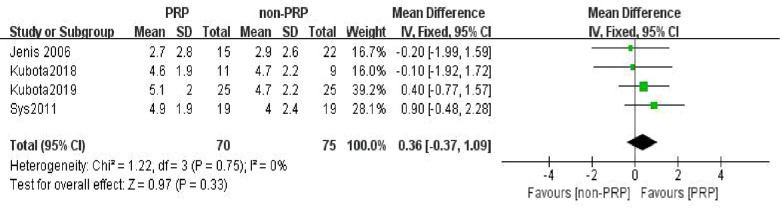
Forest plot of the VAS changes at the 24th postoperative month.

Four studies (25, 30–32) compared the VAS changes between the PRP group and the non-PRP group at the 24th postoperative month. 70 patients were included in the PRP group, and 75 patients were included in the non-PRP group. A fixed-effect model was used for meta-analysis with *I*^2^ = 0%. The outcomes indicated that there was no statistically significant difference in VAS changes between the two groups (WMD = 0.36, 95% CI: (−0.37, 1.09), *P* = 0.33) (Figure 5).

### Estimated blood loss (excluding the amount of blood consumed for PRP preparation)

**Figure 6:**
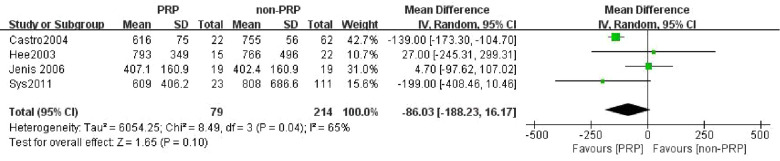
Forest plot of the EBL (excluding the amount of blood consumed for PRP preparation).

Four studies (23–25, 30) were available to merge the analysis regarding EBL, including 79 patients in the PRP group and 214 patients in the non-PRP group. A random-effects model was used for meta-analysis with *I*^2^ = 65%. The merged data showed that there was no significant difference in EBL between the two groups (WMD = −86.03, 95% CI: (−188.23, 16.17), *P* = 0.10) (Figure 6).

### Operation time

**Figure 7:**
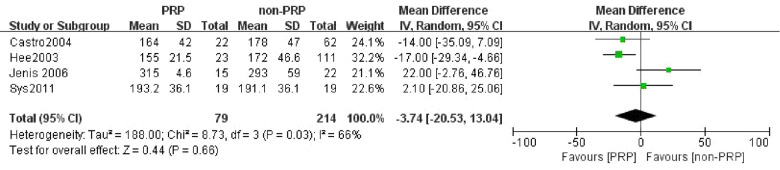
Forest plot of the operation time.

Four studies (23–25, 30) compared the operation time between the PRP group and the non-PRP group. 79 patients were in the PRP group, and 214 patients were in the non-PRP group. The random-effect model was used for meta-analysis with *I*^2^ = 66%. The outcomes indicated that there was no significant difference in operative time between the two groups (WMD = −3.74, 95% CI: (−20.53, 13.04), *P* = 0.66) (Figure 7).

### Sensitivity analysis and publication bias

**Figure 8:**
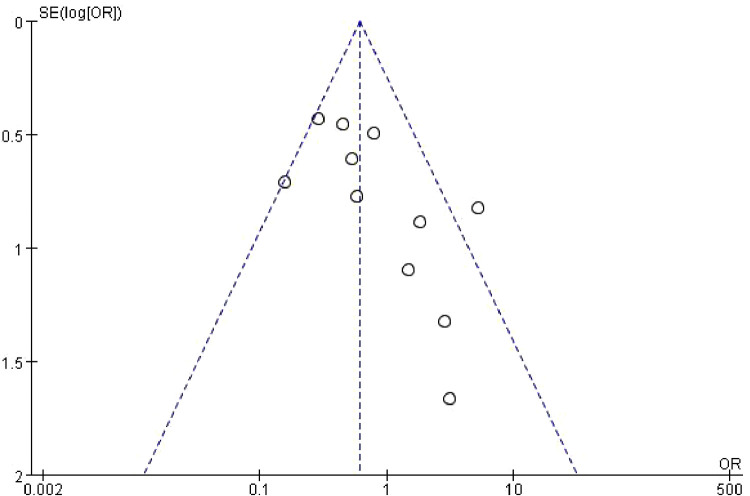
Plot of the final fusion rate.

Sensitivity analysis was performed by excluding a single study of each study and reanalyzing the data. None of the research findings showed significant changes after that analysis. Funnel plots indicated there was minimal to no bias for all included studies (Figure 8).

## Discussion

Fusion rate is considered one of the most important factors in evaluating the clinical efficacy of PRP in lumbar fusion. The clinical use of PRP in promoting spinal fusion is currently controversial. Weiner et al. (27) reasoned that PRP must have an inhibitory effect on osseointegration because PRP may interfere with the generation and function of the bone morphogenetic protein. Similar to Weiner, Castro, et al. (23) examined 22 patients who received TLIF with PRP and compared them to 62 patients who did not get PRP. They discovered no significant changes in the final lumbar fusion between the two groups, hence they did not recommend PRP for clinical use. Unfortunately, the reasons why PRP shows negative effects in clinical applications are inconclusive, and one possible explanation is that only anteroposterior radiographs are used to assess the fusion status of the spine. According to Jordan et al.'s systematic review of evaluation methods for lumbar and cervical spine fusion (3), CT and flexion-extension radiographs are the two preferred imaging modalities for determining the diagnosis of pseudarthrosis. In his description, anteroposterior radiographs had only a 43% to 82% probability of correlation with surgical exploration, with a high rate of false-negative, making them relatively insensitive in the diagnosis of pseudarthrosis (33–37). For this reason, This meta-analysis performed a subgroup analysis based on the imaging modalities used to assess spinal fusion and re-evaluated the ability of PRP promoting spinal fusion. Considering the type of bone graft (autologous, artificial, or allogeneic) may affect the activity of PRP. We also performed a subgroup analysis according to the type of grafted bone. The analysis showed that the combination of autologous bone + artificial bone may decrease the rate of spinal fusion. We all know that autologous bone has excellent osteoconductivity, osteoinductivity and osteogenesis (38). However, artificial bone or allogeneic bone is not biologically active. The implanted autologous bone provides the microporous scaffold structure required for bone growth and has good vascular growth ability, and also provides a large amount of cytokines to promote the activation of osteoblasts, making it the most ideal bone grafting material for spinal fusion (39). Our analysis suggests that the rate of spinal fusion decreased when the artificial bone is mixed with the graft material. One possible explanation is that all PRPs contain high concentrations of leukocytes (according to the PRP production process) and the potential immune response triggered by this may inhibit spinal fusion. However, this requires basic experiments to further validate. Although the subgroup analysis did not reveal that the combination of allogeneic bone + PRP inhibited spinal fusion, such result is questionable due to the small sample size (only one study).

In 2006, the concept of leukocyte-rich PRP was introduced by Everts et al. (40). Therefore, PRP can be broadly classified into two types according to the number of leukocytes contained: leukocyte-poor PRP (LP-PRP) and leukocyte-rich PRP (LR-PRP). unfortunately, none of the included literature mentioned whether the prepared PRP contained leukocytes and the concentration of leukocytes. However, the PRP preparation process was the same for all studies, i.e., the use of gravity centrifugation techniques and equipment to separate the sediment brown-yellow layer from blood units containing platelets and leukocytes, and further concentrate the brown-yellow layer (40). All of the preparation processes did not de-leukocyte the brown-yellow layer, and therefore it can be concluded that the all included studies used LR-PRP. Increased leukocyte (neutrophil) increase levels of pro-inflammatory cytokines, including interleukin-1β (IL-1β) and tumor necrosis factor-α (TNF-α), produce destructive proteases, induce an inflammatory environment, counteract the beneficial effects of growth factors, inhibit extracellular matrix secretion and promote degradation of bone and chondrocytes, impair the ability of differentiated stem cells to differentiate toward bone and cartilage, and eventually leads to degeneration of bone and cartilage tissues and aggravates the pain and swelling response (41). In contrast, the use of LP-PRP effectively reduces the concentration of inflammatory factors, decreasing the inflammatory response and accelerating tissue regeneration. A meta-analysis by Riboh et al. (42) comparing LP-PRP and LR-PRP in the treatment of knee osteoarthritis found that LP-PRP injection significantly improved the Western Ontario and McMaster Universities Osteoarthritis Index (WOMAC) and International Knee Documentation Committee (IKDC) subjective scores compared with LR-PRP or placebo. Also, the application of *P*-PRP had a lower incidence of adverse reactions compared to LR-PRP. However, there are no clinical trials assessing the efficacy of LP-PRP in promoting spinal fusion or other bone healing. Considering that currently, LR-PRP has not shown any benefit in promoting spinal fusion, LP-PRP could be a potential breakthrough.

In theory, platelet activation increases the inflammatory cascade, which may have a relieving effect on inflammatory pain. Activated platelets release many anti-inflammatory mediators that can reduce inflammation and pain. In a prospective randomized controlled trial (43), researchers evaluated the effect of applying PRP on postoperative pain reduction and functional recovery in patients who underwent open subacromial decompression, and they found patients treated with PRP demonstrated reduced visual analog scales of pain, significantly less use of pain medication, and greater shoulder range of motion compared to control patients.

Although our study did not find additional pain-relieving effects of PRP, a larger sample size is needed for further verification. Applying PRP may reduce EBL, as platelet activation is a key link in blood coagulation (25). However, our results showed that PRP can not reduce EBL. One possible explanation is that bone grafting is often the last step of spinal fusion, and most intraoperative blood loss occurs before bone grafting. It is worth noting that our estimated blood loss refers to intraoperative and postoperative blood loss and does not include the amount of blood required for PRP preparation. According to the gravity centrifugation two-step method of PRP preparation, approximately 450 ml of blood is required to make PRP; however, the concentrated red blood cell layer produced during the preparation process is perfused into the patient, so we cannot obtain the exact amount of blood loss due to PRP preparation. Given that PRP does not reduce intraoperative and postoperative blood loss, it is reasonable to believe that the total blood loss in patients in the PRP group was greater than that in the non-PRP group.

Our meta-analysis also shows that the use of PRP does not prolong the operation time (*P* = 0.66). As reported in the literature (23), anesthesia and operating room times were significantly prolonged for patients who received PRP. However, in most cases, the preparation of the PRP and its mixing with the autologous bone graft can be performed by the operating room technician, while the surgeon and his assistant can concentrate on preparing the fusion bed. therefore, the PRP preparation process does not waste too much time during the surgery.

### Limitations

We admit that the current research has limitations. To begin, while some relevant trials have been published, the number of participants in some groups was modest, and some of the research were not RCTs. Second, various confounders, such as drug use, disease history, smoking history, and primary disease were not taken into account, which adds to some heterogeneous of the pooled result, but the sensitivity analysis suggested the robustness of the results. Furthermore, there was variability in terms of follow-up time among the included study. However, a minimum follow-up period of 12 months is sufficient to observe the efficacy of PRP in spinal fusion.

## Conclusions

Compared with bone graft fusion alone, PRP cannot increase the rate of spinal fusion. Inappropriate methods of spinal fusion assessment or mixing PRP with artificial/allograft bone may have been responsible for the lower rate of spinal fusion in the PRP group.

## Data Availability

The original contributions presented in the study are included in the article/Supplementary Material, further inquiries can be directed to the corresponding author/s.
